# Overexpression of a *Malus baccata* NAC Transcription Factor Gene *MbNAC40* Increases Cold Tolerance in *Arabidopsis*

**DOI:** 10.3390/ijms27198480

**Published:** 2026-09-23

**Authors:** Kuibao Jiao, Yuze Li, Penghui Song, Wenhui Li, Zhe Zhang, Teng Wang, Xingguo Li, Deguo Han

**Affiliations:** 1 Heilongjiang Modern Agriculture Research Institute (Institute of Rural Revitalization Technology, Heilongjiang Academy of Agricultural Sciences), Harbin 150028, China; zxsgss@haas.cn (K.J.); liyz2023@163.com (Y.L.); ssph2996@163.com (P.S.); pangdun@hljsnykxyxczx.wecom.work (Z.Z.); dream525255@126.com (T.W.); 2 Key Laboratory of Biology and Genetic Improvement of Horticultural Crops (Northeast Region), Ministry of Agriculture and Rural Affairs, National–Local Joint Engineering Research Center for Development and Utilization of Small Fruits in Cold Regions, College of Horticulture & Landscape Architecture, Northeast Agricultural University, Harbin 150030, China; wenhuili@neau.edu.cn

**Keywords:** *Malus baccata* (L.) Borkh, MbNAC40, cold stress

## Abstract

As a primary apple rootstock extensively cultivated across Northeast China, *Malus baccata* (L.) Borkh. constantly suffers from low temperature, drought and salt damage throughout its growth cycle. NAC transcription factors take critical parts in coordinating plant adaptation to various adverse environments, yet relevant functional research on this woody rootstock species remains limited. In this work, a NAC family gene designated *MbNAC40* was cloned from *M. baccata*, and its biological function was explored via heterologous transformation in *Arabidopsis thaliana*. Sequence analysis revealed that the open reading frame of *MbNAC40* spans 1467 bp and encodes a 488-amino-acid protein targeted to the cell nucleus. After 24 h cold acclimation at 4 °C, plants were subjected to −4 °C freezing stress for 12 h, then recovered at 25 °C for 3 d. Transgenic *Arabidopsis* lines overexpressing *MbNAC40* exhibited much stronger cold tolerance relative to wild-type plants. Transgenic materials retained more chlorophyll and synthesized more osmoprotective proline, accompanied by enhanced activity of three antioxidant enzymes (SOD, POD and CAT). Meanwhile, multiple stress-marker genes (*AtCAT1*, *AtCSD1*, *AtAPX6*, and *AtP5CS1*) associated with ROS and osmotic adjustment were significantly activated in overexpression lines. Taken together, these results confirm that *MbNAC40* acts as a positive modulator of cold resistance in *Arabidopsis*, which provides a valuable gene resource for molecular breeding of cold-resistant *M. baccata* rootstock varieties.

## 1. Introduction

Global climate fluctuations have amplified the frequency and severity of cold incidents, imposing heavy restrictions on crop productivity and limiting the geographic cultivation range of agricultural plants [1]. Low-temperature stress disrupts cellular membrane integrity, triggers intracellular osmotic imbalance, and generates physical damage driven by ice crystal formation. Visible symptoms include retarded vegetative growth, leaf wilting, chlorosis, and severe declines in crop output and commodity quality [2,3]. To counteract cold-induced injuries, plants have evolved sophisticated multi-layered regulatory networks covering stress-signal perception, intracellular signal transduction, and genome-wide transcriptional reprogramming. As core regulatory nodes within these cascades, transcription factors (TFs) modulate downstream effector gene expression to restore cellular homeostasis and strengthen plant resistance against adverse environments [4,5].

Among diverse plant TF families, the plant-specific NAC superfamily ranks among the largest and functionally most diverse transcriptional regulators. This nomenclature originates from three founding members identified in petunia (NAM) and *Arabidopsis* (ATAF1/2, CUC2) [6]. Canonical NAC proteins feature a highly conserved N-terminal DNA-binding domain responsible for cis-element recognition and homo/heterodimer formation, while their C-terminal segments exhibit extensive length and sequence divergence, which underlies the diverse functional outputs of distinct NAC paralogs [7]. Since the landmark cloning of the petunia NAM gene in 1996 by Souer et al., accumulating research has proven NAC TFs participate in nearly all plant developmental processes. They are well-documented as governing meristem establishment and maintenance, lateral root outgrowth, secondary cell-wall biosynthesis, and leaf senescence [8]. Beyond developmental regulation, a growing number of NAC homologs participate in adaptive responses to drought, high salinity, chilling, and pathogen invasion, acting as either transcriptional activators or repressors, depending on biological contexts [9]. This dual regulatory capacity, combined with their widespread distribution across terrestrial plants, renders NAC genes central research targets for dissecting molecular modules coordinating plant growth and stress acclimation.

The ICE1-CBF-COR module constitutes the most well-characterized cold-signal transduction cascade, where NAC proteins function as vital intermediate modulators linking upstream cold signals to downstream response programs [10]. Upon cold stimulus, the MYC-type bHLH TF ICE1 undergoes activation and directly transactivates CBF family genes; accumulated CBF proteins further initiate a series of downstream transcriptional events that facilitate cold acclimation [10]. Cumulative research confirms that multiple NAC members operate either upstream or in parallel with this core pathway, exerting stimulatory or inhibitory effects on cold-signal outputs. For instance, banana *MaNAC1* acts both as a direct transcriptional target of MaICE1 and an interactive partner of MaCBF1, orchestrating cold tolerance via a dual-layer regulatory circuit [10]. Positive regulatory roles are also validated in lily and soybean: heterologous expression of *LlNAC2* elevates freezing tolerance in *Arabidopsis* through upregulating DREB1/CBF transcripts [11], whereas soybean GmNAC20 enhances cold resistance via an analogous CBF/DREB-dependent pathway [12]. In contrast, apple *MdNAC029/MdNAP* acts as a negative regulator by repressing transcription of *MdCBF1* and *MdCBF4*, ultimately weakening frost resistance [13]. NAC transcription factors also participate in cold-stress responses by regulating physiological processes such as reactive oxygen-species scavenging and osmotic homeostasis, without relying on the classical CBF pathway. Such opposite regulatory outcomes demonstrate that NAC-mediated cold signaling exhibits species-specific and context-dependent characteristics, highlighting the necessity to characterize NAC gene functions across diverse plant taxa.

Cold acclimation is not solely governed by the CBF cascade; NAC TFs also boost cold adaptability via two independent metabolic pathways, namely membrane lipid remodeling and cell-wall modification, both of which sustain cellular structural stability under low temperature. The molecular mechanism of lipid metabolism regulation has been thoroughly illustrated in banana: *MaNAC25* and *MaNAC28* jointly activate a suite of phospholipase genes including *MaPLDa1/4*, *MaPLDb1/2/3*, *MaPLDd1/2/5*, *MaDGK3* and *MaPLC1/2*. Coordinated activation of these genes raises cellular phosphatidic acid (PA) concentrations, forming a positive feedback loop that aggravates chilling damage. Notably, ethylene mitigates such harmful consequences by suppressing the transcriptional activity of both NAC regulators [14]. A comparable regulatory pattern exists in pepper, where CaNAC1 directly induces *CaPLDa4* expression, accelerating membrane lipid degradation and reducing cold resilience [15]. Apart from lipid turnover, lignin biosynthesis represents another cell-wall-associated defensive strategy against cold stress. In loquat fruit, EjXND1 physically interacts with the lignin-promoting transcription factor EjHB1 and attenuates its transactivation capacity toward *EjPRX12*, thereby fine-tuning lignin deposition levels [16]. Conversely, EjNAC3 confers positive cold protection by directly activating *EjCAD*, a rate-limiting gene in lignin biosynthetic pathways [17]. Collectively, these findings reveal that NAC TFs coordinate multi-tiered regulatory networks, integrating CBF-dependent transcriptional cascades with separate lipid and cell-wall metabolic pathways to orchestrate cold adaptation across different plant species.

Mounting evidence validates the indispensable roles of NAC TFs in cold-stress responses across various plant species. Nevertheless, functional investigations focusing on woody perennial plants, especially those endemic to frigid winter zones, remain relatively scarce. *Malus baccata*, a deciduous Rosaceae tree naturally distributed across cold, seasonally arid regions of Northeast China, Mongolia and Siberia, serves as an ideal experimental material for such stress-resistance research [18]. Apart from its ornamental value and applications in tea processing and wood carving, this species is widely adopted as apple rootstock, due to its exceptional tolerance to freezing, drought and saline soils [19]. Recognizing its value as a repository of elite stress-tolerant genetic resources, our research team previously cloned and characterized two NAC homologs from *M. baccata*, *MbNAC25* and *MbNAC29*, both of which positively modulate cold and salt resistance [20,21]. Building upon these prior findings, the present study reports the isolation and functional identification of *MbNAC40*, an uncharacterized NAC gene from the same woody species. Through systematic bioinformatic prediction, multi-stress expression profiling and heterologous transformation in *Arabidopsis*, we verify the fact that *MbNAC40* functions as a positive effector of cold tolerance. Our results not only expand the inventory of functionally validated NAC genes mediating abiotic-stress responses in woody plants, but also provide promising genetic material for molecular breeding of cold-resistant *M. baccata* rootstock varieties in future research.

## 2. Results

### 2.1. Molecular Cloning and Bioinformatic Profiling of MbNAC40

Sequence decoding of the cloned fragment revealed that the complete coding sequence (CDS) of *MbNAC40* spanned 1467 base pairs and encoded a deduced polypeptide consisting of 488 amino acid residues (Figure S1). Computational prediction of its physicochemical properties yielded the molecular formula C_2378_H_3679_N_727_O_741_S_19_, a molecular weight of 54.918 kDa, and a theoretical isoelectric point (pI) of 6.57. This protein contained 52 positively charged and 61 negatively charged amino acids. Its grand average of hydropathicity (GRAVY) value was calculated as −0.930, alongside an instability index of 53.04, demonstrating that MbNAC40 is a hydrophilic protein which is unstable in vitro.

The prediction of secondary structural components illustrated that random coils dominated the MbNAC40 polypeptide, accounting for 83.4% of the total sequence, whereas extended strands and α-helices only made up 7.58% and 9.02%, separately (Figure S2A). A three-dimensional homologous structural model was constructed via homology-based simulation (Figure S2B), whose structural composition matched the secondary-structure prediction results well. A canonical NAM conserved domain was mapped to amino acid residues 47-188 (Figure S2C), which verified MbNAC40 as a bona fide member of the NAC TF family. Multiple sequence alignment further corroborated this classification result (Figure 1A). Phylogenetic reconstruction grouped MbNAC40 into the same evolutionary clade with a PbNAC75-like homologous protein from *Pyrus bretschneideri*, implying a close evolutionary affinity between these two proteins (Figure 1B).

### 2.2. Subcellular Localization of MbNAC40 Protein

Transient agroinfiltration in epidermal cells of *Nicotiana benthamiana* leaves was performed to clarify the subcellular compartment where MbNAC40 functions. Two constructs, the fusion vector 35S::MbNAC40-GFP and empty control 35S::GFP, were independently delivered into tobacco leaf tissue. Laser-scanning confocal microscopic observation displayed ubiquitous green fluorescent signals across the whole cell in the empty GFP control group. In contrast, fluorescence derived from MbNAC40-GFP fusion protein exclusively accumulated within cell nuclei (Figure 2). Such imaging evidence confirms that MbNAC40 is a nucleus-targeted transcription factor.

### 2.3. Tissue and Stress-Induced Expression Patterns of MbNAC40 in Malus baccata

RT-qPCR quantification was carried out to dissect the transcriptional variation of *MbNAC40* across various plant tissues and multiple abiotic-stress treatments, aiming to uncover its potential roles in developmental regulation and stress adaptation. Tissue-expression profiling demonstrated that the transcript abundance of *MbNAC40* peaked in the new leaf (Figure 3A).

Given its distinct expression characteristics, we selected the new leaf and stem for further investigation of their time-dependent expression under four abiotic-stress conditions: drought, salt, cold, and heat. These stresses elicited different transcriptional response patterns. For new-leaf samples under salt treatment, gene expression stayed nearly unchanged at the 1 h time point, rose sharply after 0, 1, 3, 6, 9 and 12 h of treatment, and reached the maximum level at 6 h, showing an approximate 2.4-fold elevation relative to untreated controls. Drought stress triggered steady, continuous transcriptional induction from 1 h post-treatment, with the highest expression (~4.0-fold higher than CK) detected at 6 h. Cold stimulation induced fast and robust activation of *MbNAC40*: transcript levels were significantly elevated as early as 1 h, kept rising thereafter, and hit a peak at 9 h, with an over 10-fold increase. Hot treatment also significantly boosted gene expression at 6 h, with the peak value reaching roughly 2.3-fold that of the control level (Figure 3B).

Stem tissues exhibited vastly different temporal expression trends compared with the new leaf. Both salt and osmotic stresses induced maximal transcription at 3 h, with respective induction folds of ~2.5 and ~2.4. Under heat treatment, the highest transcript accumulation appeared at 9 h (approximately 2.6-fold), while cold stress triggered a rapid, short-term expression surge at 1 h, upregulating transcription by around 4.2 times compared with untreated samples (Figure 3C). Among all four stress treatments, cold exposure generated the strongest transcriptional activation of *MbNAC40*, which drove us to further explore its biological function in cold resistance.

### 2.4. Heterologous Overexpression of MbNAC40 Improves Cold Resistance in Transgenic Arabidopsis

To validate whether *MbNAC40* participates in plant cold acclimation, we generated transgenic *Arabidopsis* plants constitutively expressing *MbNAC40* driven by the CaMV 35S promoter via the agrobacterium-mediated floral-dip transformation method. A total of six independent T3 homozygous transgenic lines were obtained in this study. To ensure the statistical reliability of phenotypic assays and the representativeness of molecular detection, we selected three independent high-expression homozygous lines (L1, L3, L5) for subsequent functional verification. Three homozygous T_3_ transgenic lines (L1, L3, L5) with remarkably high *MbNAC40* transcript levels were screened out for subsequent functional phenotype tests (Figure 4A).

Under standard growth conditions without cold treatment, WT and transgenic *Arabidopsis* exhibited indistinguishable morphological phenotypes. After 24 h cold acclimation at 4 °C, plants were subjected to −4 °C freezing stress for 12 h, then recovered at 25 °C for 3 d; however, severe leaf wilting and tissue damage were observed in WT plants, whereas transgenic lines maintained intact leaf morphology with minor injury (Figure 4B). After three days of recovery cultivation at 25 °C, survival statistics showed that the mortality rate of WT seedlings was more than twice that of the three overexpression lines (Figure 4C).

Physiological biomarker detection provided solid supporting evidence for the enhanced cold adaptability of *MbNAC40*-overexpressing plants. Following cold stress, transgenic lines retained far more chlorophyll than WT controls (Figure 5A), while the osmoprotectant proline accumulated to significantly higher content in overexpression materials (Figure 5B). The enzymatic activities of three core antioxidant enzymes (SOD, POD, and CAT) were all markedly higher in transgenic plants, compared with WT (Figure 5C–E). Furthermore, under the same cold conditions, the electrolyte leakage (a widely used indicator of cell membrane integrity) in the *MbNAC40* overexpression strain was significantly lower than that in the WT strain (Figure 5F). Taken together, these physiological data illustrate that *MbNAC40* confers enhanced cold resistance in *Arabidopsis* through three major pathways: sustaining photosynthetic pigment stability, mitigating membrane lipid peroxidation, and strengthening the plant antioxidant defense system.

### 2.5. Transcriptional Alterations of Cold-Responsive Marker Genes in Transgenic Arabidopsis

To uncover the molecular regulatory mechanism underlying *MbNAC40*-mediated cold tolerance, we quantified the transcript abundance of several well-documented stress-marker genes in both untreated and cold-challenged *Arabidopsis* seedlings. As illustrated in Figure 6, freezing treatment drastically upregulated four functional genes (*AtCSD1*, *AtCAT1*, *AtAPX6*, and *AtP5CS1*) in *MbNAC40* overexpression lines relative to WT plants. This coordinated transcriptional activation suggests that MbNAC40 may be associated with the removal of reactive oxygen species and the expression of homeostatic genes within cells, further modulating plant cold tolerance.

## 3. Discussion

NAC TFs act as core regulatory hubs governing plant adaptive reactions against adverse environments, and abundant literature has validated their critical functions in coping with abiotic stresses [22,23]. Benefiting from rapid innovations in genome sequencing, NAC family genes have been thoroughly explored in herbaceous model crops including rice [24], *Arabidopsis* [25] and tobacco [26]. Nevertheless, relevant research on non-model woody species is still far insufficient. Cold stress disturbs intracellular steady-state balance via multiple detrimental pathways, such as triggering membrane lipid peroxidation, suppressing photosynthetic efficiency and causing membrane stability [22]. In the present study, a cold-activated NAC gene *NAC40* was cloned from *Malus baccata*. Subsequent structural and evolutionary characterization combined with heterologous functional verification verified the fact that overexpression of this gene can remarkably strengthen plant cold resistance.

Bioinformatic identification validated the fact that MbNAC40 harbors a typical NAM conserved domain (Figure S1) and targets cell nuclei (Figure 2), two defining characteristics shared by all NAC transcription factor members [27]. Evolutionary tree analysis grouped MbNAC40 together with a PbNAC75-like homologous protein derived from *Pyrus bretschneideri* (Figure 1). This close genetic relationship indicates sequence similarity between the two proteins, which provides sequence-level clues for further functional investigation of NAC genes across different plant taxa. Such sequence conservation also supports the hypothesis that NAC proteins carry out analogous stress-modulating roles within woody Rosaceae plants, and implies MbNAC40 functions as an evolutionarily conserved stress-responsive modulator in *M. baccata*.

Tissue-expression screening illustrated the fact that the transcriptional level of *MbNAC40* peaked in the new leaf, which hints at its potential participation in photosynthetic tissue growth or stress-signal transduction within leaf organs. Tissue-biased expression is a widespread feature among NAC homologs across various plant taxa. For example, two celery NAC genes, *AgNAC63* and *AgNAC47*, display dominant expression in leaf tissues [28], while multiple radish *RsNAC* transcripts accumulate preferentially in roots and leaf [29]. Furthermore, the transcription of *MbNAC40* was drastically stimulated by cold, drought, salt, and hot stimuli, with unique time-dependent expression fluctuations under each stress condition (Figure 3). Such broad stress inducibility aligns with the universal capacity of NAC proteins to coordinate plant adaptation to various abiotic threats [30]. Previous research has reported similar multi-stress-responsive NAC members: 18 leaf-specific and 17 root-specific stress-inducible NAC genes were screened in sugar beet, separately [31]; celery *AgNAC63* can be strongly activated by heat, cold and salt treatments, simultaneously [28]; and transcriptome profiling of grain amaranth also uncovered multiple *AhypNAC* genes with prominent upregulation upon abiotic challenges [32]. Overall, these expression features solidly connect *MbNAC40* to abiotic-stress regulatory pathways and lay a solid groundwork for deeper mechanistic exploration of this gene, consistent with our previous studies on other NAC homologs from *M. baccata* [20,21].

NAC TFs have emerged as key regulators in plant cold signaling, and their functional mechanisms have attracted increasing attention. In *Ammopiptanthus mongolicus*, *AmNAC24* enhances cold tolerance in transgenic *Arabidopsis* by maintaining cellular redox homeostasis [33]. Similarly, *HuNAC20* and *HuNAC25* from pitaya (*Hylocereus undatus*) improve cold resistance through upregulating cold-responsive genes such as *AtRD29A* and *AtCOR15A* [34]. In apple, *MdNAC104* functions through both CBF-dependent and CBF-independent pathways to modulate cold tolerance [35]. Likewise, *SINAC90* in tomato and *CaNAC035* in pepper—the latter induced by both cold and ABA—have been characterized as positive regulators of cold-stress responses [36,37]. In line with these findings, our study demonstrated that overexpression of *MbNAC40* in *Arabidopsis* significantly increased survival rates following cold stress, indicating that *MbNAC40* acts as a positive regulator of cold tolerance (Figure 4 and Figure 5). Collectively, these results not only confirm the functional conservation of stress-responsive NAC transcription factors across diverse plant species, but also underscore the value of mining stress-tolerance genes from cold-hardy woody germplasm native to northeastern China.

Based on the above research results, the subsequent work will conduct in-depth analysis of the regulatory mechanism and application potential of *MbNAC40* from the following three aspects. Firstly, at the molecular regulation level, yeast two-hybrid library screening and ChIP-seq techniques will be used to identify the interacting proteins of MbNAC40 and its downstream target genes, thereby constructing the signal transduction network of *MbNAC40* in the response to cold stress. Secondly, at the biological function level, stable transgenic materials of *M. baccata* will be constructed, and the stress-resistance function of *MbNAC40* will be systematically evaluated under natural overwintering conditions, providing phenotypic evidence for its practical application in woody plants. Finally, at the breeding application level, functional molecular markers based on *MbNAC40* will be developed and applied in the molecular breeding practice of *M. baccata* for stress resistance, providing genetic resources and technical support for the cultivation of new varieties of rootstocks resistant to cold.

## 4. Materials and Methods

### 4.1. Plant Materials, Growth Conditions and Treatments

*M. baccata* seedlings were obtained from the 726 Research Group, College of Horticulture, Northeast Agricultural University. *Arabidopsis thaliana* ecotype Col-0 was used in this study. Seeds were surface-sterilized and sown on 1/2 MS medium. After stratification at 4 °C for 2 d, seedlings were sown in a 3:1 (*v*/*v*) mixture of nutrient soil and vermiculite, with 8 plants per 7 cm diameter pot at 22 °C/20 °C (day/night), with a 16 h light/8 h dark photoperiod, light intensity of 120 μmol m^−2^ s^−1^, and relative humidity maintained at 60–70%. 60-day-old tissue-cultured plants of uniform size and vigor were grown at 25 °C as the control, while parallel groups were subjected to four stress treatments: cold (4 °C), salt (200 mM NaCl), drought (20% PEG6000), and heat (37 °C) [38]. For time-course analysis, samples were collected immediately at 0, 1, 3, 6, 9 and 12 h. In addition, various tissues were collected from normally growing plants maintained at 25 °C [39]. All samples were immediately frozen in liquid nitrogen and maintained at −80 °C until use.

### 4.2. Cloning and Bioinformatic-Expression Analysis of MbNAC40

Based on the apple *MdNAC40* reference sequence (GenBank: MG099877), gene-specific primers *MbNAC40*-F/R were designed (Supplementary Table S2). Using KOD high-fidelity DNA polymerase, the *MbNAC40* CDS was amplified from *M. baccata* cDNA and inserted into pEasy-T5 Zero. The resulting constructs were transformed into competent cells, with transformants selected on kanamycin (50 mg/L) plates after overnight incubation at 37 °C. Colony PCR screening combined with Sanger sequencing was implemented, to authenticate positive recombinant clones, referring to the experimental workflow reported previously [20]. Sequence identity between MbNAC40 and MdNAC40 was calculated via DNAMAN 5.2, with 96.93% amino acid identity and 97.41% CDS nucleotide identity. MbNAC40 were kindly provided by Dr. Li (Key Laboratory of Biology and Genetic Improvement of Horticultural Crops, Ministry of Agriculture and Rural Affairs, National–Local Joint Engineering Research Center for Development and Utilization of Small Fruits in Cold Regions, College of Horticulture, Northeast Agricultural University, Harbin, China).

All bioinformatic analyses were performed to decode the intrinsic features of the MbNAC40 protein. The ExPASy ProtParam online platform was adopted to compute fundamental physicochemical properties of the deduced amino acid sequence. Conserved functional domains were further screened by searching the NCBI Conserved Domain Database (CDD). Sequence-homology alignment between MbNAC40 and its orthologs from various plant species was completed with DNAMAN 5.2 software, and a Neighbor-Joining phylogenetic tree was constructed in MEGA 11.0 with 1000 bootstrap repetitions for reliability assessment. The SOPMA server was utilized to predict the secondary structural components, including α-helices, β-sheets and random coils. Meanwhile, the SWISS-MODEL platform was applied to build the three-dimensional homologous structural model of MbNAC40 [19]. Detailed links to all online bioinformatics tools are listed in Table S1. Cis-element analysis of the 2 kb promoter regions of *AtCSD1*, *AtCAT1*, *AtAPX6* and *AtP5CS1* was performed. Several putative cis-elements harboring the CACG core NAC-recognition sequence were detected (Supplementary Table S3), implying potential binding of NAC transcription factors to these promoters.

### 4.3. Vector Construction

For construction of the overexpression recombinant vector, two specific primers, *MbNAC40*-2F and *MbNAC40*-2R, were synthesized with *BamH* I and *Sal* I restriction-enzyme cleavage sites incorporated at their terminals (Table S2). The complete coding sequence of *MbNAC40* was amplified by PCR, followed by agarose gel separation, target-fragment excision and purification. The backbone vector pCAMBIA2300 (35S::GFP) was linearized and ligated to the recovered target fragment through homologous recombination. The ligation product was introduced into competent Escherichia coli DH5α cells, and transformants were cultured on kanamycin-supplemented solid medium, for screening. Candidate positive recombinants were further authenticated by Sanger sequencing to obtain the final expression vector 35S::MbNAC40-GFP, as previously described [39]. Correctly sequenced plasmids were delivered into Agrobacterium tumefaciens GV3101 competent cells, via electroporation. Positive agrobacterial strains were screened using colony PCR and reserved for subsequent plant genetic transformation and subcellular-localization detection experiments.

### 4.4. Subcellular Localization of MbNAC40

Transient expression assays in *Nicotiana benthamiana* leaves were adopted to identify the subcellular distribution of MbNAC40 protein. The Agrobacterium tumefaciens GV3101 strain carrying the recombinant 35S::MbNAC40-GFP vector was cultured until the OD_600_ value reached 1.6. Bacterial precipitates were collected via centrifugation and resuspended in infiltration buffer (10 mM MES, 10 mM MgCl_2_, 0.2 mM acetosyringone), with the final OD_600_ adjusted to 0.4. The prepared bacterial solution was injected into abaxial epidermal cells of fully unfolded tobacco leaves, using a needle-free syringe. After infiltration, treated tobacco plants were incubated in dark conditions at 25 °C for 48 h, to facilitate sufficient expression of the fusion protein. Fluorescent signals derived from GFP were visualized with a Leica TCS SP8 laser scanning confocal microscope (Wetzlar, Germany); the excitation wavelength was set at 488 nm, and the emission signal was captured at 525 nm [40].

### 4.5. Reverse Transcription–Quantitative PCR (RT-qPCR) Analysis of MbNAC40

Total RNA extraction was performed on all plant samples collected in Section 4.1 with TRIzol reagent supplied by Tiangen Biotech (Beijing, China). Genomic DNA contamination was eliminated in the reverse-transcription procedure using a gDNA removal kit from Tiangen Biotech (Beijing, China). Specific primer pairs for RT-qPCR amplification were designed with SnapGene 6.0.2 software, and their sequences are listed in Supplementary Table S2. The 20 μL quantitative PCR reaction system consisted of 2× SYBR Green master mix (TransGen Biotech), 0.2 μM forward and reverse primers, as well as 1 μL 10-fold diluted cDNA template. Amplification reactions were carried out on a Bio-Rad CFX96 real-time PCR instrument, according to the thermal cycling program: an initial 2 min denaturation step at 95 °C, followed by 40 cycles of 5 s at 95 °C and 30 s annealing at 60 °C. The housekeeping gene *MbAct* was selected as the internal reference for transcript normalization. The relative expression level of target genes was quantified via the 2^−ΔΔCt^ algorithm as, reported previously [25], and the expression value of control samples was normalized to 1.0 as the baseline.

### 4.6. Plant Transformation

Genetic transformation of wild-type (WT) *Arabidopsis* was implemented via the floral-dipping method mediated by Agrobacterium tumefaciens harboring the 35S::MbNAC40-GFP recombinant vector. Prior to infection, GV3101 agrobacteria carrying the target construct were collected through centrifugation, then resuspended in infiltration solution supplemented with 5% sucrose and 0.04% Silwet L-77. The inflorescences of 5–6 week-old *Arabidopsis* were immersed in the bacterial mixture for 30–60 s, with mild shaking. Post-transformation, these plants were covered with transparent plastic covers to sustain high humidity and incubated overnight under dark environments, followed by cultivation under regular growth regimes.

After seed maturation, T_1_ seeds were gathered and surface-disinfected using 10% sodium hypochlorite solution for 10 min. Sterile water was applied to fully wash away residual disinfectant, and seeds were placed on 1/2 MS solid medium supplemented with 50 mg/L kanamycin to screen positive transgenic individuals. Seedlings with green cotyledons and intact true leaves under kanamycin pressure were preliminarily judged as potential transgenic lines.

To screen stable homozygous strains, mature T_1_ plants were self-pollinated to harvest T_2_ seeds, which underwent identical kanamycin screening procedures. T_3_ progenies exhibiting complete kanamycin resistance without trait segregation were identified as homozygous transgenic lines. RT-qPCR detection was further adopted to validate positive homozygotes: total RNA was extracted from leaf samples and reverse-transcribed into cDNA templates. Transcript abundance of *MbNAC40* was quantified using specific primer pairs listed in Table S2, with *AtActin* serving as the reference gene for normalization. Ultimately, three independent homozygous lines with abundant *MbNAC40* transcripts were chosen for subsequent phenotypic observation and physiological index measurement.

### 4.7. Physiological Measurements

A cold-exposure experiment was carried out to evaluate frost-resistance performance of four-week-old WT *Arabidopsis* and three *MbNAC40* overexpression lines. The whole cold-treatment procedure contained three successive stages: a 24 h cold-acclimation period at 4 °C, a 12 h freezing challenge at −4 °C without gradual cooling, and a three-day recovery phase at 25 °C, under a standard photoperiod (16 h light/8 h dark). Digital images of plant morphology were captured at 0, 1, 3, 6, 9 and 12 h after treatment to record phenotypic differences, and plant survival percentages were statistically calculated afterwards.

Rosette leaf samples were harvested right after cold treatment for subsequent physiological parameter detection. The chlorophyll and proline contents, as well as the enzyme activities of SOD (BC5165), POD (BC0090) and CAT (BC0205), were detected using commercial kits (Solarbio, Beijing, China). All the operation steps were carried out strictly in accordance with the instructions. In addition, the electrolyte leakage rate was measured by an electrical conductivity meter.

### 4.8. Expression Analysis of Stress-Related Genes

Leaf samples were harvested from WT *Arabidopsis* and T_3_ transgenic lines before and after cold treatment, following the sampling scheme detailed in Section 2.5. Total RNA was isolated from these materials and reverse-transcribed into cDNA with the identical reaction system mentioned in Section 2.5. RT-qPCR was implemented to measure the transcriptional abundance of four stress-related genes, namely *AtCSD1* (NC_003070.9), *AtCAT1* (NM_101914.4), *AtAPX6* (NM_119384.4) and *AtP5CS1* (NM_129539.2). The constitutively expressed *AtActin* gene was selected as the reference standard to normalize target gene expression levels. Sequences of all primer pairs applied in this assay are listed in Table S2.

### 4.9. Statistical Analysis

Every experimental group was set up with three separate biological replicates, and each biological sample contained three technical replicates for repeated detection. Raw measurement data were preliminarily sorted in Microsoft Excel. Statistical comparison was conducted via one-way analysis of variance (ANOVA), and significant differences between groups were further examined using Tukey’s multiple comparison test in SPSS 20.0 software (IBM Corporation, Armonk, NY, USA). One-way ANOVA followed by Tukey’s post hoc test was performed to compare physiological and phenotypic parameters between genotypes at each identical cold-treatment time point. Differences with *p* < 0.05 were defined as statistically significant. All quantitative data in this study are presented as mean ± standard error (SE) from three independent biological replicates.

## 5. Conclusions

In summary, we cloned the *MbNAC40* ortholog from *Malus baccata* and functionally verified its positive role in cold tolerance via heterologous overexpression in *Arabidopsis thaliana*. The encoded protein carries a conserved NAM domain, localizes to the nucleus, accumulates preferentially in the new leaf, and responds strongly to cold stimulation. Ectopic expression of *MbNAC40* in *Arabidopsis* not only improves cold survival rates but is also associated with elevated transcription levels of genes corresponding to various stresses. Thus, a working model can be proposed: low-temperature-induced MbNAC40 is associated with downstream genes related to ROS detoxification and osmotic homeostasis, thereby protecting membrane function and photosynthetic efficiency under low temperatures (Figure 7). Our findings position *MbNAC40* as a promising genetic resource for enhancing cold hardiness in *M. baccata* rootstocks and other woody perennials.

## Figures and Tables

**Figure 1:**
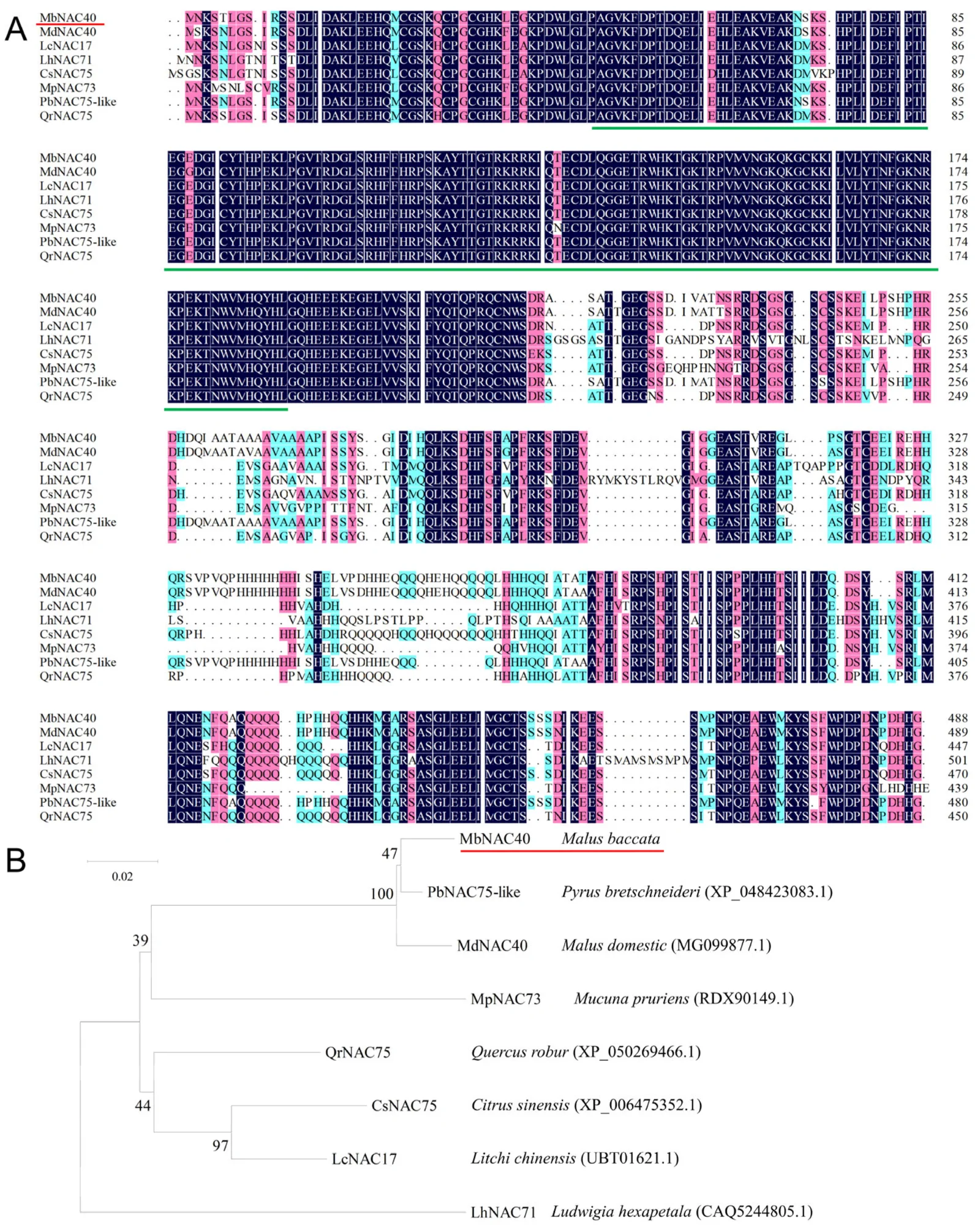
MbNAC40 homology analysis. (**A**) Multiple sequence alignment of MbNAC40 protein. The green line indicates the NAC domain. (**B**) Phylogenetic tree of MbNAC40 protein. The red underlining is the MbNAC40 protein.

**Figure 2:**
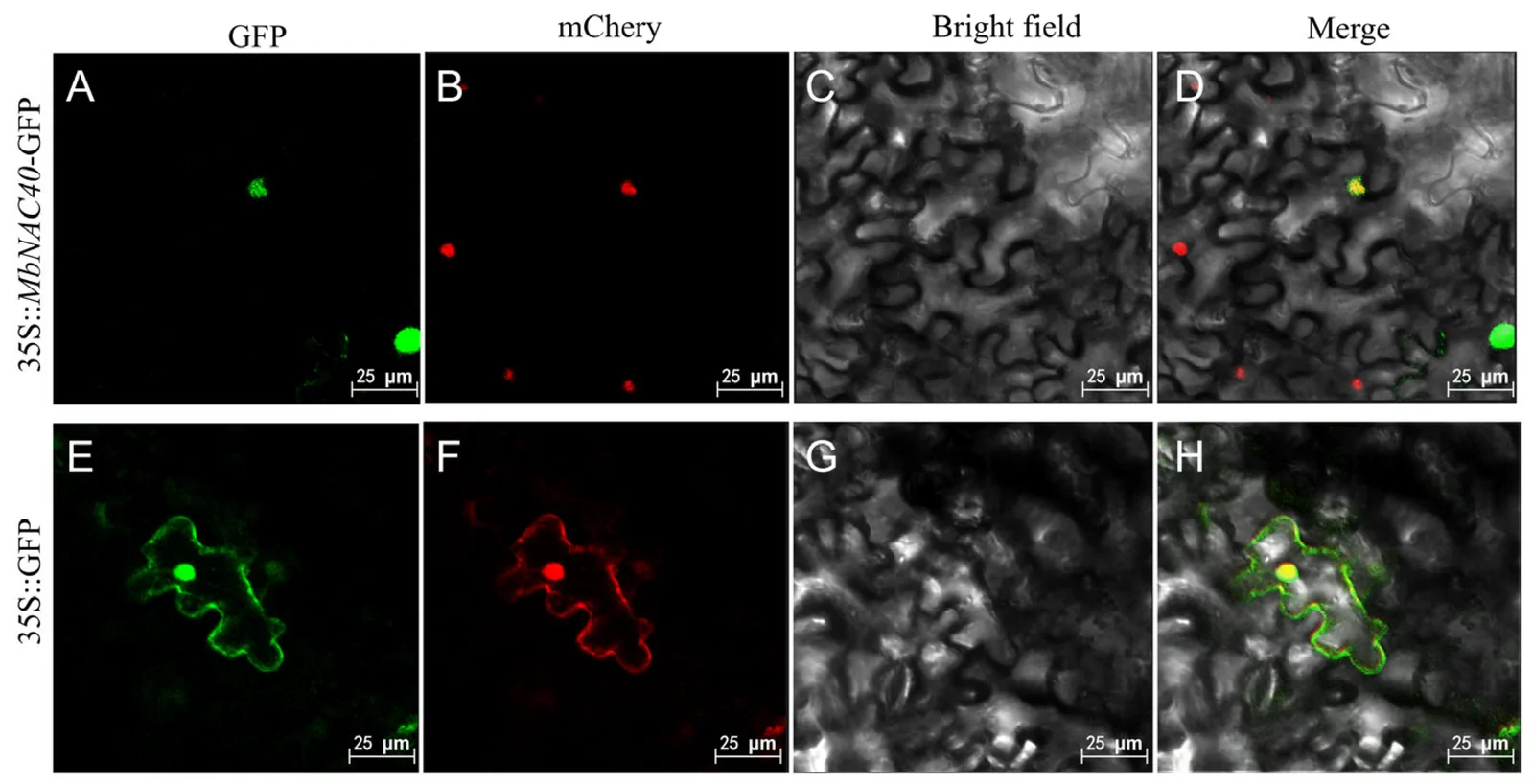
Subcellular localization. (**A**,**E**) GFP channel displays the green fluorescence signals images of 35S::GFP and 35S::MbNAC40-GFP. (**B**,**F**) The fluorescence signal images of Marker represents the signal of nucleus. (**C**,**G**) Images of cells to be detected under bright light. (**D**,**H**) Image merge. Bar = 25 μm.

**Figure 3:**
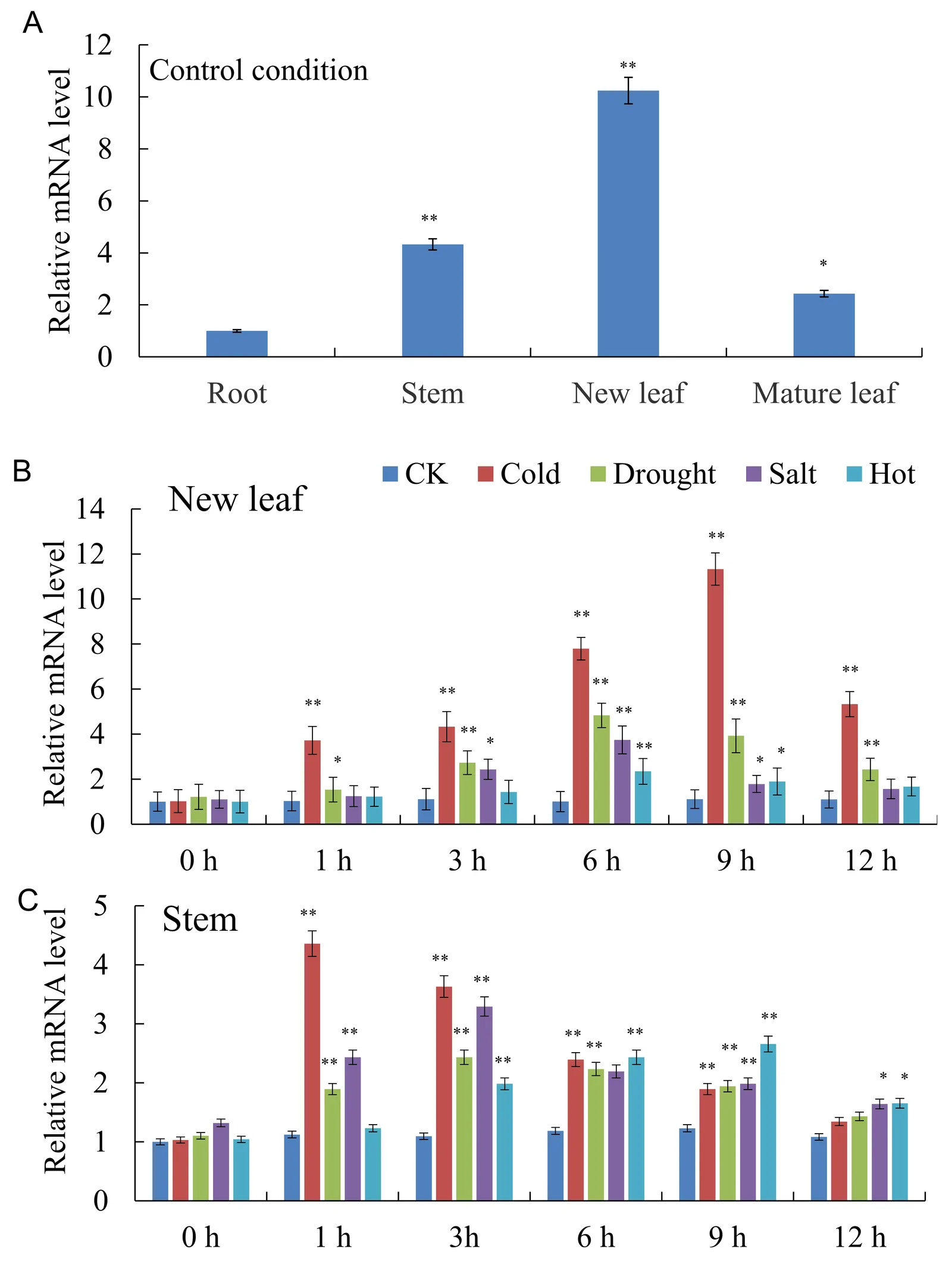
Expression of the *MbNAC40* gene in *M. baccata*. (**A**) Tissue-specific expression patterns of *MbNAC40*. Take root as control. (**B**) The expression levels of *MbNAC40* in new leaf and (**C**) stem under different adverse treatments. Take stress treatments for 0 h as the control (CK), and select *MbAct* as the internal reference control. Each value is the mean ± SE of three independent determinations and asterisks indicate significant differences at * *p* ≤ 0.05, ** *p* ≤ 0.01.

**Figure 4:**
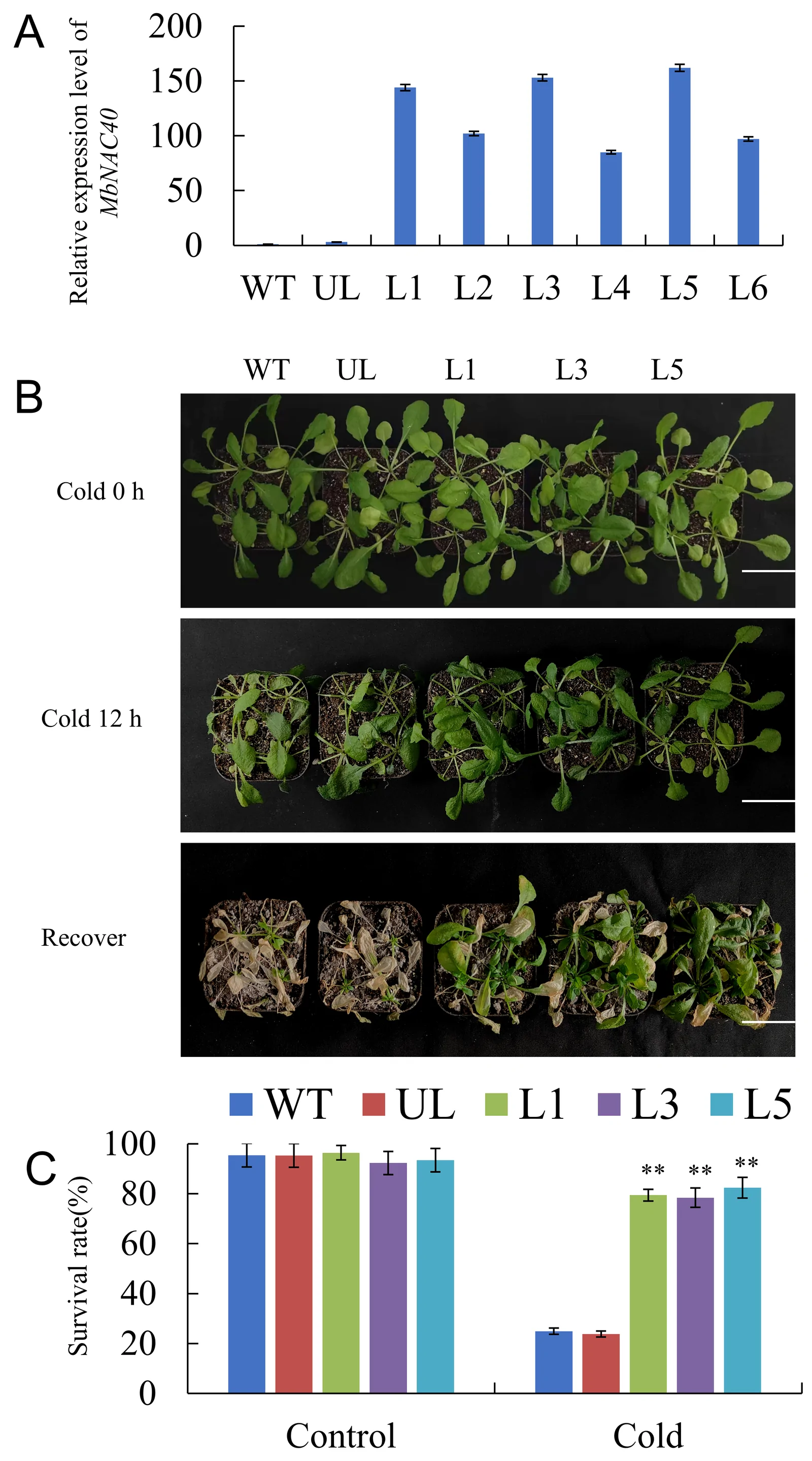
Phenotypes and survival rates of different strains of *Arabidopsis* under cold. (**A**) Relative expression level of *MbNAC40* in *Arabidopsis*. (**B**) Phenotypes (Cold 0 h, 12 h, and recover to normal growth). The white scale represents 3 cm. (**C**) Survival rates of different lines (control = Cold 0 h, and 12 h). Each value is the mean ± SE of three independent determinations and asterisks indicate significant differences at ** *p* ≤ 0.01. “UL” is the negative control with the same genetic background, only transformed with an empty vector without the target gene.

**Figure 5:**
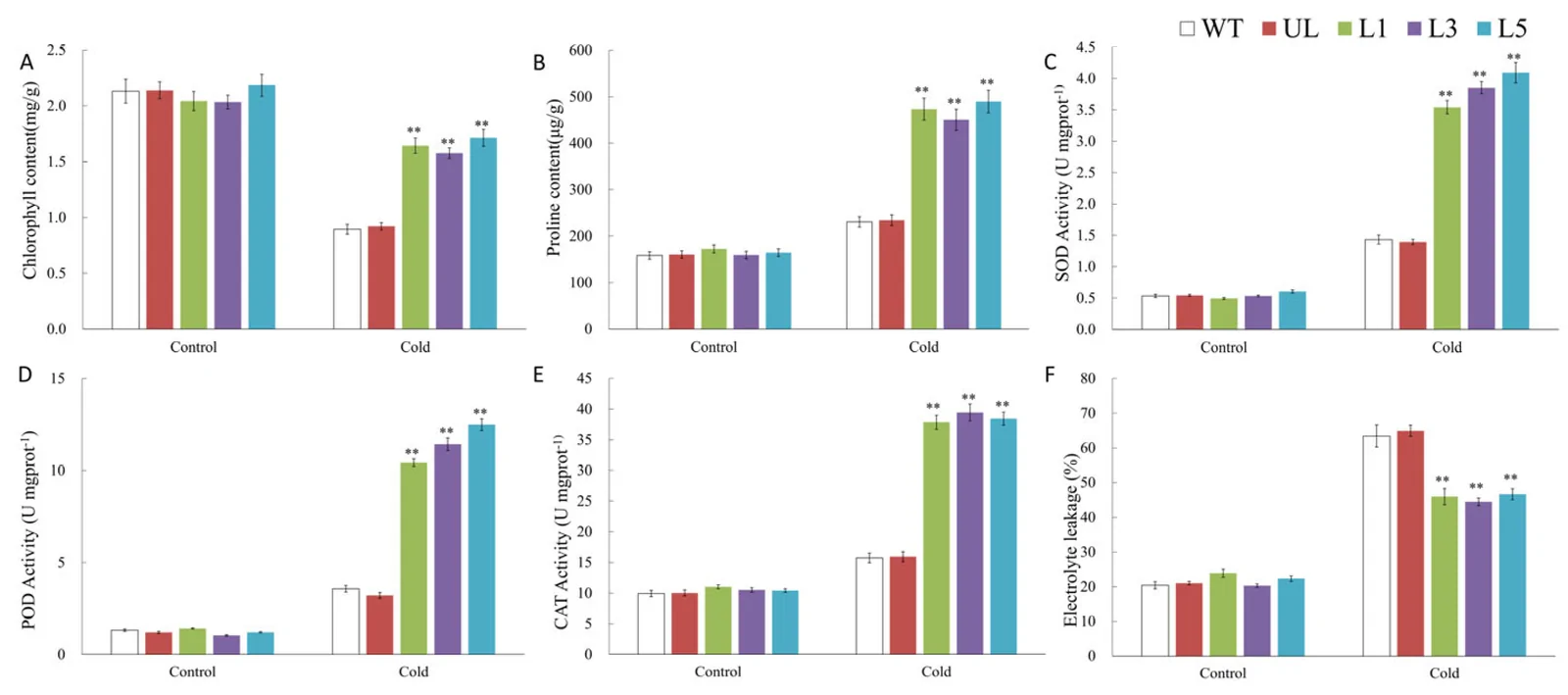
Evaluation of physiological indices responsive to cold stress. The contents of (**A**) chlorophyll and (**B**) proline; the activities of (**C**) SOD, (**D**) POD, (**E**) CAT and (**F**) EL. Each index of WT was used as the control. Each value is the mean ± SE of three independent determinations and asterisks indicate significant differences at ** *p* ≤ 0.01. “UL” is the negative control with the same genetic background, only transformed with an empty vector without the target gene.

**Figure 6:**
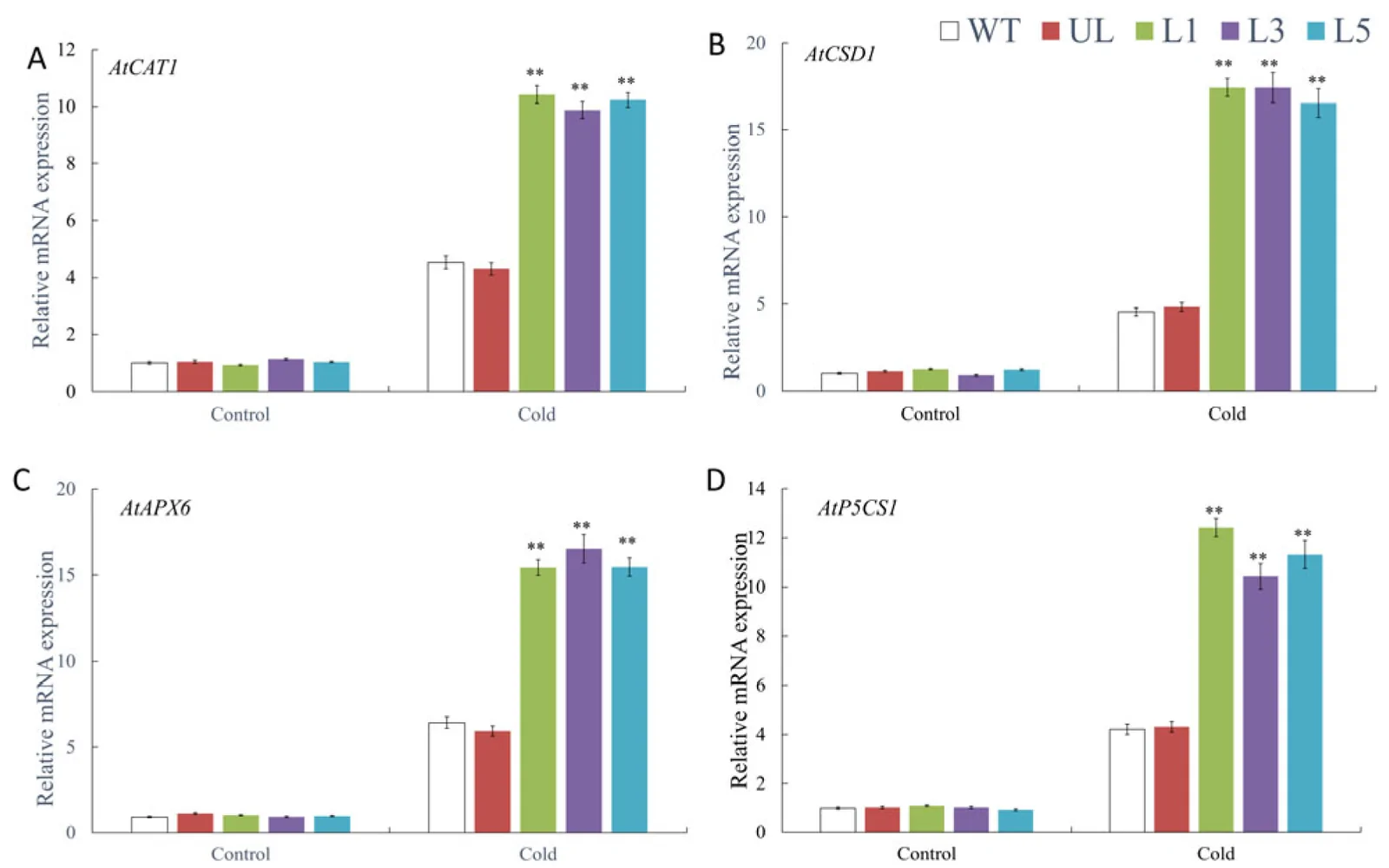
RT-qPCR analyses of the (**A**) *AtCAT1*, (**B**) *AtCSD1*, (**C**) *AtAPX6* and (**D**) *AtP5CS1* genes under cold stress in *Arabidopsis* leaves. Each value is the mean ± SE of three independent determinations, and asterisks indicate significant differences at ** *p* ≤ 0.01.

**Figure 7:**
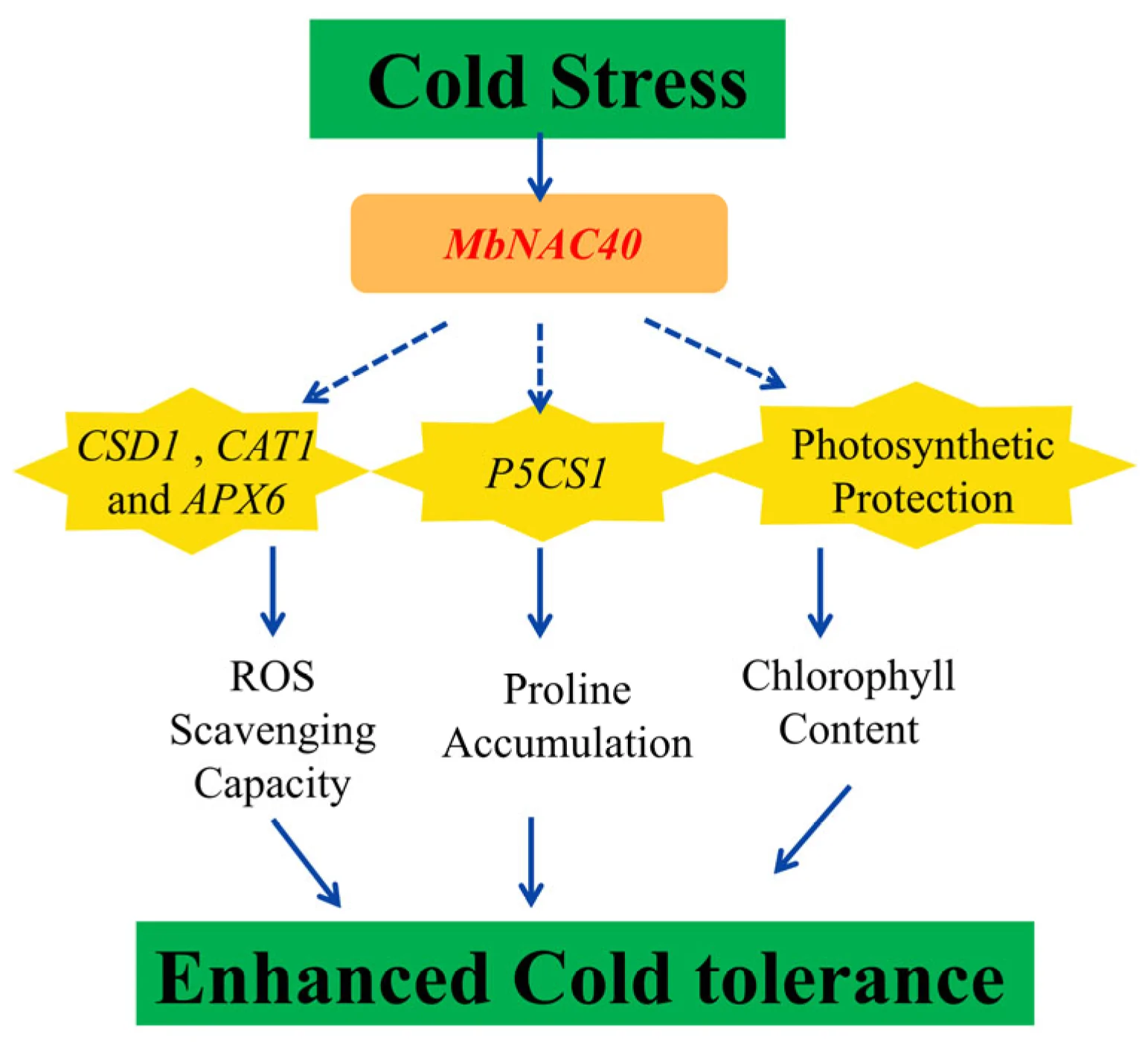
The schematic diagram of *MbNAC40* mechanism under cold stresses is speculated. Dashed line: expression correlation.

## Data Availability

Data is contained within the article and Supplementary Materials.

## Supplementary Materials

The following supporting information can be downloaded at: https://www.mdpi.com/article/10.3390/ijms27198480/s1.
