# Supplementary material for: Overexpression of a Malus baccata NAC Transcription Factor Gene MbNAC40 Increases Cold Tolerance in Arabidopsis
Source: Int J Mol Sci. 2026 Sep 23;27(19):8480. doi: 10.3390/ijms27198480 (PMC13664912; doi:10.3390/ijms27198480)
Supplement: Supplementary file 1 [file ijms-27-08480-s001.zip › supplementary figure.pdf]

A

```
1    ATGAATAAGAGTACTTTGGGGTCCATCAGAAGTTCTGATCTCATTGATGCAAAGCTTGAAGAGCATCAGATGTGCGGATCCAAGCAGTGTCCCGGTTGTGGACACAAACTCGAAGGAAAGCCGGACTGGTTAGGTCTACCAGCAGGA
1     M  N  K  S  T  L  G  S  I  R  S  S  D  L  I  D  A  K  L  E  E  H  Q  M  C  G  S  K  Q  C  P  G  C  G  H  K  L  E  G  K  P  D  W  L  G  L  P  A  G

148  GTGAAATTTGATCCAACAGACCAAGAACTAATTGAACACCTTGAAGCAAAGGTAGAAGCCAAAAACTCCAAGTCTCACCCTTTGATTGATGAATTCATCCCTACAATTGAAGGAGAAGATGGAATTTGTTACACCCATCCTGAAAAA
50    V  K  F  D  P  T  D  Q  E  L  I  E  H  L  E  A  K  V  E  A  K  N  S  K  S  H  P  L  I  D  E  F  I  P  T  I  E  G  E  D  G  I  C  Y  T  H  P  E  K

295  CTTCCAGGAGTCACAAGAGATGGCTTGAGCAGGCACTTCTTCCATAGACCTTCAAAAGCCTACACAACTGGGACAAGAAAGAGAAGAAAAATCCAAACAGAATGTGACTTGCAAGGTGGGGAAACAAGATGGCACAAGACAGGCAAG
99    L  P  G  V  T  R  D  G  L  S  R  H  F  F  H  R  P  S  K  A  Y  T  T  G  T  R  K  R  R  K  I  Q  T  E  C  D  L  Q  G  G  E  T  R  W  H  K  T  G  K

442  ACCAGGCCAGTCATGGTGAATGGAAAACAAAAAGGGTGCAAGAAAATTCTAGTCCTCTACACCAACTTTGGCAAAAACCGAAAACCCGAAAAGACGAATTGGGTGATGCACCAATACCATCTTGGGCAGCATGAGGAGGAGAAAGAA
148   T  R  P  V  M  V  N  G  K  Q  K  G  C  K  K  I  L  V  L  Y  T  N  F  G  K  N  R  K  P  E  K  T  N  W  V  M  H  Q  Y  H  L  G  Q  H  E  E  E  K  E

589  GGAGAGCTTGTGGTGTCAAAGATATTCTATCAGACTCAGCCAAGGCAATGCAACTGGTCTGATAGAGCGAGTGCAACTGGTGAAGGAAGCAGTGATATTGTGGCTACTAATAGCAGAAGAGATAGTGGGAGTGGGAGTTGTTCTTCT
197   G  E  L  V  V  S  K  I  F  Y  Q  T  Q  P  R  Q  C  N  W  S  D  R  A  S  A  T  G  E  G  S  S  D  I  V  A  T  N  S  R  R  D  S  G  S  G  S  C  S  S

736   AAGGAAATATTACCCTCTCATCCTCATAGAGATCATGACCAAATAGCTGCAACAGCAGCAGCTGCGGTTGCAGCTGCTGCTCCAATCTCAAGCTACAGTGGCATTGACATTCATCAGTTGAAGTCAGATCACTTCAGCTTTGCCCCC
246   K  E  I  L  P  S  H  P  H  R  D  H  D  Q  I  A  A  T  A  A  A  A  V  A  A  A  A  P  I  S  S  Y  S  G  I  D  I  H  Q  L  K  S  D  H  F  S  F  A  P

883  TTCAGAAAAAGCTTTGATGAGGTGGGGATAGGAGGAGAGGCTTCAACTGTAAGGGAAGGGCTGCCATCTGGCACGTGCGAAGAGATACGAGAGCACCACCAGAGATCAGTACCAGTACAGCCTCATCATCACCATCATCATCATATA
295   F  R  K  S  F  D  E  V  G  I  G  G  E  A  S  T  V  R  E  G  L  P  S  G  T  C  E  E  I  R  E  H  H  Q  R  S  V  P  V  Q  P  H  H  H  H  H  H  H  I

1030 AGCCATGAACTAGTACCCGATCATCACGAGCAGCAGCAGCAACATGAACATCAACAGCAGCAGCAACTGCATCATCATCAACAAATTGCAACAGCTACAGCTTTCCACATCAGCCGCCCTTCACATCCCATTTCCACCATCATCTCT
344   S  H  E  L  V  P  D  H  H  E  Q  Q  Q  Q  H  E  H  Q  Q  Q  Q  Q  L  H  H  H  Q  Q  I  A  T  A  T  A  F  H  I  S  R  P  S  H  P  I  S  T  I  I  S

1177 CCACCTCCTCTCCACCACACGTCCATCATTCTTGATCAGGACTCCTACTCCCGGTTGATGCTCCAAAACGAAAATTTCCAGGCGCAGCAACAGCAGCAACACCCCCACCACCAACAGCATCATAAAATGGGAGCTAGGTCTGCATCT
393   P  P  P  L  H  H  T  S  I  I  L  D  Q  D  S  Y  S  R  L  M  L  Q  N  E  N  F  Q  A  Q  Q  Q  Q  Q  H  P  H  H  Q  Q  H  H  K  M  G  A  R  S  A  S

1324 GGTTTGGAGGAACTAATAATGGGCTGCACTTCCAGCAGTAGTGATATCAAAGAAGAGTCATCGATGCCAAACCCTCAAGAGGCAGAGTGGATGAAGTACTCCTCCTTCTGGCCTGACCCTGACAACCCAGATCATCATGGGTAG
442   G  L  E  E  L  I  M  G  C  T  S  S  S  S  D  I  K  E  E  S  S  M  P  N  P  Q  E  A  E  W  M  K  Y  S  S  F  W  P  D  P  D  N  P  D  H  H  G  *
```

B

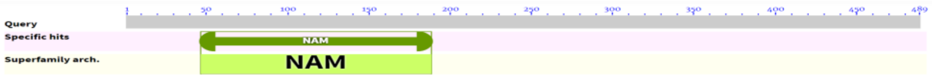

Name	Accession	Description	Interval	E-value
NAM	pfam02365	No apical meristem (NAM) protein	47-188	1.49×10^{-42}

Figure S1: Sequence analysis of MbNAC40 protein. **(A)** Amino acid sequences of MbNAC40. **(B)** Conserved domain of MbNAC40 protein.

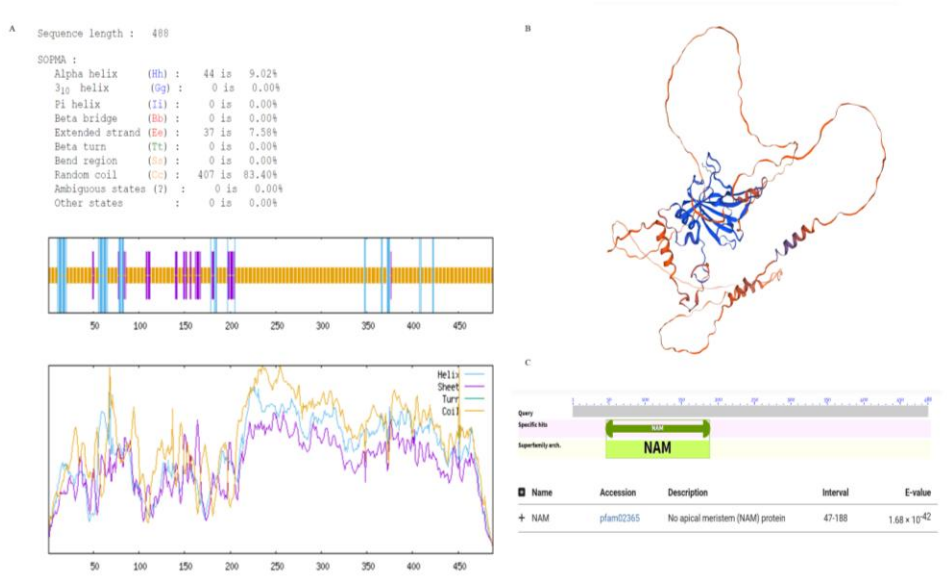

Figure S2: Secondary structure and tertiary structure prediction of MbNAC40 protein. **(A)** MbNAC40's structural analysis at the secondary level. **(B)** Tertiary structural prediction of MbNAC40. **(C)** Conserved structural domain study of MbNAC40.
