# Supplementary material for: Overexpression of a Malus baccata NAC Transcription Factor Gene MbNAC40 Increases Cold Tolerance in Arabidopsis
Source: Int J Mol Sci. 2026 Sep 23;27(19):8480. doi: 10.3390/ijms27198480 (PMC13664912; doi:10.3390/ijms27198480)
Supplement: Supplementary file 1 [file ijms-27-08480-s001.zip › Supplementary Table.pdf]

Table S1. The specific URLs of the websites used in this article.

Name	Website addresses
ExPASy	https://web.expasy.org/cgi-bin/protparam/protparam
NCBI	https://www.ncbi.nlm.nih.gov/
SOPMA	https://npsa.lyon.inserm.fr/cgi-bin/secpred_sopma.pl
SWISS-MODEL	https://swissmodel.expasy.org/

Table S2. Primers used in this study.

Primer Name	Primer sequence (5'→3')
MbNAC40-F/R	ATGAATAAGAGTACTTTGGGGTC/CCCATGATGATCTGGGTTGTC
MbNAC40-2F/2R	GGACAGGGTACCCGGGGATCCATGAATAAGAGTACTTTGGGGTC/CTGGCATGCCTGCAGGTCGACCCCATGATGATCTGGGTTGTC
MbNAC40-qF/R	ACTGGGACAAGAAAGAG/CAAAGTTGGTGTAGAGG
MbAct-F/R	GGATTCTGGTGATGGTGT/CCACCGATCCAGACACTG
AtActin-F/R	AGCACAACAGGCGGGAAAG/AGGCATTCTTGTTGGTCTCTGACAT
AtCSD1-F/R	AGTGAGGGTGTTACGGG/GGGGTTGAAATGTGGAC
AtCAT1-F/R	GAGGAGCCAATCACAGC/AAGACCAAGCGACCAAC

AtAPX6-F/R	CTACCTCCAGAAACCCT/CCTTTACTCCCAATCGT
AtP5CS1-F/R	CTTTACACAGGCCCTCC/GTCATACCCCCTCTCCC

Table S3. Putative cis-elements containing the CACG core NAC-recognition motif in the 2-kb promoter regions of AtCSD1, AtCAT1, AtAPX6 and AtP5CS1

**Gene**	**Locus ID**	**NCBI Accession**	**Predicted cis-element**	**Motif sequence (containing CACG core)**	**Position (relative to ATG)**	**Strand**
AtCSD1	AT1G08830	NC_003070.9	G-Box	CACGTT	357	+
AtCSD1	AT1G08830	NC_003070.9	G-box	CACGTC	1325	-
AtCSD1	AT1G08830	NC_003070.9	ABRE4	CACGTA	1365	+
AtCAT1	AT1G20630	NM_101914.4	ABRE	GACACGTGGC	1197	-
AtCAT1	AT1G20630	NM_101914.4	G-box	CACGTC	1199	+
AtCAT1	AT1G20630	NM_101914.4	G-Box	CACGTT	1380	-
AtCAT1	AT1G20630	NM_101914.4	ABRE4	CACGTA	1792	+
AtCAT1	AT1G20630	NM_101914.4	ACE	GACACGTATG	1858	-
AtCAT1	AT1G20630	NM_101914.4	ABRE4	CACGTA	1860	-
AtP5CS1	AT2G39800	NM_129539.2	ABRE	GACACGTGGC	1808	+
AtP5CS1	AT2G39800	NM_129539.2	G-box	ACACGTGGC	1809	+
AtP5CS1	AT2G39800	NM_129539.2	G-Box	CACGTG	1810	-
AtP5CS1	AT2G39800	NM_129539.2	G-box	CACGTG	1810	-
AtP5CS1	AT2G39800	NM_129539.2	ABRE	CACGTG	1810	-
AtAPX6	AT4G32320	NM_119384.4	G-box	CACGAC	514	-
